# Epidemiology and Clinical Profile of Cutaneous Warts in Chinese College Students: A Cross-Sectional and Follow-Up Study

**DOI:** 10.1038/s41598-018-33511-x

**Published:** 2018-10-18

**Authors:** Jianjun Liu, Hongtian Li, Fan Yang, Yingyun Ren, Tianbao Xia, Zigang Zhao, Xiaojia Cao, Zekun Wang, Mengmeng Yin, Shichao Lu

**Affiliations:** 1 Department of Dermatology, 306 Hospital of PLA, Beijing, 100101 China; 2 Institute of Reproductive and Child Health/Ministry of Health Key Laboratory of Reproductive Health, Peking University Health Science Center, Beijing, 100191 China; 3 Department of Dermatology, The Second People’s Hospital of Wuqing, Tianjin, 301700 China; 4 Department of Dermatology, The Third People’s Hospital of Foshan, Foshan, 528000 China; 5 Department of Dermatology, Chinese PLA General Hospital, Beijing, 100853 China

## Abstract

In this study, the hands and feet of 15,384 undergraduate and postgraduate students in 3 colleges in Beijing were examined for the presence of cutaneous warts at college-entry, and those diagnosed with warts were followed up 2–3 years later. We identified totally 215 (1.4%; 95% CI, 1.2–1.6%) students with warts. The prevalence was significantly higher in male than in female students (2.0% *vs*. 0.9%, *P* < 0.0001). Of the 215 patients, 66.9% and 62.1% had only one wart and 98.3% and 93.2% had warts <1 cm in diameter, on the hands and feet, respectively. Of the 130 patients with a follow-up visit, 78 did not receive any treatment (44 recovered within 2 years). Patients aged 21–25 compared to those aged ≤20 were more likely to be free of warts (hazard ratio = 1.76; 95% CI, 1.07–2.89), while lower father’s education (hazard ratio = 0.19; 95% CI, 0.04–0.98) and poor sleep quality (hazard ratio = 0.41; 95% CI, 0.18–0.92) decreased the likelihood of resolution. The prevalence of warts is 1.4% in college students. The majority of patients have warts <1 cm and approximately 2/3 patients has one wart. Slightly over half of patients recover spontaneously within 2 years. Patients’ age, sleep quality, and paternal education may affect the resolution.

## Introduction

Cutaneous warts are caused by the human papilloma virus (HPV), the most prevalent types of which are HPV2/27/57 and HPV1^1–7^. While most patients with cutaneous warts are asymptomatic, some may experience physical or psychological discomfort^8^. Many studies have documented the prevalence of cutaneous warts in children^9–13^, ranging widely from 3.3% in the USA to 33% in the Netherlands^11,13^. Data on the prevalence of cutaneous warts in young adults are limited compared with those in children. In an earlier UK study with a modest sample size, the prevalence was reported to be 3.5% in people aged 25 to 34 years and 0.3% in those aged 35 to 54 years^14^. In another earlier US study with a relatively large sample size, the prevalence of wart was 1.2% in people aged 18 to 24 years and 0.6% in those aged 25 to 34^15^. Neither of these reports provided data on the determinants and clinical profile of warts in adults^14,15^.

Cutaneous warts may resolve spontaneously. Bruggink *et al*. found that one-half of primary schoolchildren with warts would be free of warts within one year, and young age and non-Caucasian skin type were protective factors for wart resolution^16^. In comparison to children, the natural course and prognostic factors of warts in adults are scarce, although these information may be of particular importance in shared decision-making with patients.

In this study, we aimed to determine the prevalence, clinical profile, and prognosis of cutaneous warts in Chinese college students, a typical young adult population. We also aimed to identify potential factors influencing the occurrence and resolution of warts.

## Results

### Prevalence of Cutaneous Warts

Of the 15,384 students, 215 were diagnosed with cutaneous warts, with an overall prevalence of 1.4% (95% CI, 1.2–1.6%). The prevalence was significantly higher in male than in female students (2.0% *vs*. 0.9%, *P* < 0.0001) and in students from rural than those from urban areas (1.7% *vs*. 1.3%, *P* = 0.03). The prevalence tended to be negatively associated with maternal and paternal education levels (*p* for trend test = 0.070 and 0.014, respectively). No statistical difference was observed in the prevalence of cutaneous warts between groups defined by participants’ age, ethnicity, region, or parents’ occupation. In the multivariate logistic regression analysis, male gender was associated with an increased risk of warts (aOR = 2.06; 95% CI, 1.55–2.74) (Table 1). Further analyses performed separately for subtypes of cutaneous warts showed that male gender was associated with an increased risk of both common (aOR = 2.38; 95% CI, 1.60–3.55) and plantar warts (aOR = 2.04; 95% CI, 1.32–3.14).

**Table 1:** Univariate and Multivariate Logistic Regression of Potential Factors Associated With the Prevalence of Warts (n = 15384).

Characteristics	No. of cases/No. of students (%)^a^	*P* value	Unadjusted OR (95% CI)	Adjusted OR (95% CI)^b^
Age, years				
14–20	119/8249 (1.4)		1.00 (ref)	1.00 (ref)
21–25	83/6185 (1.3)		0.94 (0.71,1.24)	0.94 (0.70, 1.26)
26–35	13/811 (1.6)		1.12 (0.63, 2.00)	1.03 (0.55, 1.92)
36–44	0/74 (0.0)	0.68	<0.001 (<0.001, >999)	<0.001 (<0.001, >999)
Sex				
Female	83/8830 (0.9)		1.00 (ref)	1.00 (ref)
Male	132/6461 (2.0)	<0.0001	2.20 (1.67, 2.90)	2.06 (1.55, 2.74)
Ethnicity				
Han	190/13789 (1.4)		1.00 (ref)	1.00 (ref)
Other	24/1458 (1.7)	0.41	1.20 (0.78, 1.84)	1.09 (0.69, 1.71)
Residential area				
Urban	133/10427 (1.3)		1.00 (ref)	1.00 (ref)
Rural	81/4705 (1.7)	0.03	1.37 (1.03, 1.81)	1.19 (0.79, 1.80)
Region				
North	58/4463 (1.3)		1.00 (ref)	1.00 (ref)
Northeast	32/1692 (1.9)		1.46 (0.95, 2.26)	1.51 (0.96, 2.37)
East	45/3598 (1.3)		0.96 (0.65, 1.42)	0.91 (0.61, 1.36)
Central	23/2105 (1.1)		0.84 (0.52, 1.36)	0.79 (0.48, 1.30)
South	7/610 (1.1)		0.88 (0.40, 1.94)	0.87 (0.39, 1.93)
Northwest	24/1134 (2.1)		1.64 (1.02, 2.65)	1.45 (0.88, 2.38)
Southwest	22/1515 (1.5)		1.12 (0.68, 1.84)	1.05 (0.63, 1.74)
Hong Kong, Macao, and Taiwan	0/44 (0.0)	0.16	<0.001 (<0.001, >999)	<0.001 (<0.001, >999)
Father’s occupation				
Farmer	69/4276 (1.6)		1.00 (ref)	1.00 (ref)
Worker	41/2533 (1.6)		1.00 (0.68, 1.48)	1.42 (0.76, 2.65)
Clerk	64/5300 (1.2)		0.75 (0.53, 1.05)	1.15 (0.58, 2.31)
Other	41/3129 (1.3)	0.28	0.81 (0.55, 1.20)	1.16 (0.59, 2.29)
Mother’s occupation				
Farmer	79/4785 (1.7)		1.00 (ref)	1.00 (ref)
Worker	29/1972 (1.5)		0.89 (0.58, 1.37)	0.83 (0.42, 1.65)
Clerk	61/5013 (1.2)		0.73 (0.52, 1.03)	0.96 (0.47, 1.94)
Other	46/3469 (1.3)	0.31	0.80 (0.56, 1.15)	0.95 (0.49, 1.84)
Father’s education level				
High school above	78/6429 (1.2)		1.00 (ref)	1.00 (ref)
High school	59/3979(1.5)		1.23 (0.87, 1.72)	0.94 (0.60, 1.46)
Junior school	59/3810 (1.6)		1.28 (0.91, 1.80)	0.93 (0.56, 1.55)
Less than junior school	19/1073 (1.8)	0.32	1.47 (0.89, 2.43)	0.78 (0.39, 1.59)
Mother’s education level				
High school above	60/5279 (1.1)		1.00 (ref)	1.00 (ref)
High school	54/3808 (1.4)		1.25 (0.86, 1.81)	1.21 (0.77, 1.90)
Junior school	58/3951 (1.5)		1.30 (0.90, 1.86)	1.25 (0.74, 2.11)
Less than junior school	43/2289 (1.9)	0.09	1.67 (1.12, 2.47)	1.53 (0.83, 2.82)

^a^Numbers may not add up to total due to missing data. ^b^Mutually adjusted for all other variables in the table. CI = confidence interval; OR = odds ratio.

### Clinical Characteristics of Warts

Of the 215 patients diagnosed with cutaneous warts, 52.1% had common warts; 40.5%, plantar warts; 5.1%, plane warts; 1.7%, plantar as well as common warts; and 0.5%, plane as well as common warts (Table 2). Of the 212 patients with records on location of warts, 10.4% had wart(s) on the dorsum of the left hand; 19.3%, on the dorsum of the right hand; 14.2%, on the sole of the left foot; 17.0%, on the sole of the right foot; 10.4%, on any two of the aforementioned locations; 28.8%, on other non-common locations (Table 2). Of the 126 patients with warts on the hands, 66.9% had a single wart, and 98.3% had warts <1 cm in diameter; the corresponding percentages were 62.1% and 93.2%, for the 103 patients with warts on the feet, respectively (Table 2). Forty-nine patients (22.8%) had visited a doctor.

**Table 2:** Clinical Characteristics of the Patients With Warts (n = 215).

Characteristics	Frequency (n)^a^	%^b^
Type of warts		
Common wart only	112	52.1
Plantar wart only	87	40.5
Plane wart only	11	5.1
Common wart as well as plantar	4	1.7
Common wart as well as plane	1	0.5
Location of warts		
Dorsum of the left hand	22	10.4
Dorsum of the right hand	41	19.3
Sole of the left foot	30	14.2
Sole of the right foot	36	17.0
Any two of the aforementioned locations	22	10.4
Other non-common locations	61	28.8
No. of warts on the hands^c^		
1	79	66.9
2	13	11.0
3–4	12	10.2
5–9	8	6.8
10 or more	6	5.1
No. of warts on the feet^c^		
1	64	62.1
2	13	12.6
3-4	7	6.8
5–9	11	10.7
10 or more	8	7.8
Size of warts on the hands^c^		
<1 cm	116	98.3
≥1 cm	2	1.7
Size of warts on the feet^c^		
<1 cm	96	93.2
≥1 cm	7	6.8
Predisposing factors		
Friction	42	19.5
Trauma	23	10.7
Direct skin-to-skin contact	2	0.9
Any two of the aforementioned factors	4	1.9
Unknown	144	67.0
Discomfort caused by warts		
No	186	86.5
Worry of infection	12	5.6
Affecting walking, sports, and work	10	4.7
Unsightly appearance	4	1.9
Any two of the aforementioned discomfort	3	1.4
Symptoms		
No	181	84.2
Itching	8	3.7
Pain during activity	24	11.2
Spontaneous pain	1	0.5
Pain during activity as well as spontaneous pain	1	0.5

^a^Numbers may not add up to total due to missing data. ^b^Sum of percentages may not total 100 due to rounding off. ^c^Of the 215 patients, 112 had warts on the hands, 97 had warts on the feet, and 6 had warts on both the hands and feet.

Of the 215 patients, 71 (33.0%) reported one or more suspected predisposing factors of warts, including friction (42 patients, 19.5%), trauma (23 patients, 10.7%), direct skin-to-skin contact (2 patients, 0.9%), and any two of the aforementioned factors (4 patients, 1.9%) (Table 2). Of the 215 patients, 29 (13.5%) reported to have suffered from discomfort caused by the warts, including worrying of infection (5.6%), affecting walking, sports and work (4.7%), unsightly appearance (1.9%), and any two of the aforementioned discomfort (1.4%) (Table 2). Thirty-four patients (15.8%) reported to have had clinical symptoms, including itching (3.7%), pain during activity (11.2%), spontaneous pain (0.5%), and both pain during activity and spontaneous pain (0.5%) (Table 2). The self-reported scores on pain intensity during activity, as assessed by a 0 (no pain) to 10 (worst pain imaginable) point numeric rating scale, ranged from 1 to 5 points (mean ± SD, 3.1 ± 1.5).

### Follow-up Results

In total, 130 (60.5%) of the 215 patients received a follow-up survey: 95 were re-examined by a trained dermatologist on site, and 35 were interviewed via telephone. Of the 85 patients who were lost to follow-up, 60 patients could not be contacted and 25 refused to participate. All demographic and clinical characteristics, except father’s education level, did not differ between patients who participated in the follow-up survey and those who did not (Table 3).

**Table 3:** Comparison of Characteristics Between Patients Who Participated in the Follow-up Survey and Those Who did not.

Characteristics	Not participated (n = 85)^a^	Participated (n = 130)	*Χ* ^2^	*P* value
Age, years				
≤20	40	79		
21–25	38	45		
≥26	7	6	4.215	0.122
Sex				
Male	52	80		
Female	33	50	0.003	0.957
Residential area				
Urban	47	86		
Rural	37	44	2.257	0.133
Father’s occupation				
Farmer	31	38		
Worker	19	22		
Clerk	19	45		
Other	16	25	4.235	0.237
Mother’s occupation				
Farmer	37	42		
Worker	9	20		
Clerk	24	37		
Other	15	31	3.562	0.313
Father’s education level				
High school above	24	54		
High school	27	32		
Junior school	22	37		
Less than junior school	12	7	8.024	0.046
Mother’s education level				
High school above	20	40		
High school	24	30		
Junior school	24	34		
Less than junior school	17	26	1.592	0.661
Smoking				
Yes	3	7		
No	81	123	0.080	0.778
Drinking				
Yes	24	40		
No	58	90	0.054	0.817
Regular exercise				
Yes	54	79		
No	30	51	0.268	0.605
Poor sleep quality				
Yes	11	15		
No	73	115	0.116	0.734
High psychological stress				
Yes	22	25		
No	63	105	1.331	0.249
Type of warts				
Common wart only	49	63		
Plantar wart only	29	58		
Other^b^	7	9	2.351	0.309
Number of warts				
Single	57	74		
Multiple	26	56	2.955	0.086

^a^Numbers may not add up to total due to missing data. ^b^Other types include common as well as plantar wart, common as well as plane wart, and plane wart only.

Of the 130 patients, 78 did not receive any treatment, of whom 24 (30.8%) recovered spontaneously within 1 year, 20 (25.6%) between 1 and 2 years, and 10 (12.8%) beyond 2 years. Of the 52 who had received treatments, 26 (50.0%) were treated with cryotherapy, 7 (13.5%) with CO_2_ laser, 2 (3.8%) with both cryotherapy and1064-nm long-pulsed Nd: YAG laser, 5 (9.6%) with plasters containing 78% salicylic acid and 4% phenol, 3 (5.8%) with traditional Chinese medicine, 1 (1.9%) with interferon-α gel, 1 (1.9%) with retinoids cream, and 7 (13.5%) with unclear treatments.

### Factors Influencing Resolution of Warts

In the multivariate analyses, the middle age group (aged 21 to 25 years *vs*. ≤20 years) was a protective factor for the resolution of warts, whereas lower father’s education level and poor sleep quality were risk factors for the resolution (Table 4).

**Table 4:** Univariate and Multivariate Cox Regression Analyses of Potential Factors Affecting Resolution of Warts (n = 130).

Characteristics	Total No. of patients (n)	No. of patients with complete resolution (%)	Crude HR (95% CI)	Adjusted HR (95% CI)^a^
Age, years				
≤20	79	57 (72.2)	1.00 (ref)	1.00 (ref)
21–25	45	36 (80.0)	1.38 (0.91–2.10)	1.76 (1.07–2.89)
≥26	6	3 (50.0)	0.57 (0.18–1.82)	0.64 (0.18–2.31)
Sex				
Female	50	34 (68.0)	1.00 (ref)	1.00 (ref)
Male	80	62 (77.5)	1.25 (0.82–1.91)	1.41 (0.78–2.55)
Residential area				
Urban	86	63 (73.3)	1.00 (ref)	1.00 (ref)
Rural	44	33 (75.0)	1.03 (0.68–1.57)	0.87 (0.42–1.80)
Father’s occupation				
Farmer	38	30 (79.0)	1.00 (ref)	1.00 (ref)
Worker	22	19 (86.4)	1.02 (0.58–1.82)	1.30 (0.35–4.85)
Clerk	45	30 (66.7)	0.74 (0.45–1.24)	0.65 (0.17–2.50)
Other	25	17 (68.0)	0.80 (0.44–1.46)	0.93 (0.24–3.61)
Mother’s occupation				
Farmer	42	33 (78.6)	1.00 (ref)	1.00 (ref)
Worker	20	16 (80.0)	0.88 (0.48–1.59)	0.55 (0.16–1.95)
Clerk	37	27 (73.0)	0.85 (0.51–1.42)	1.11 (0.32–3.85)
Other	31	20 (64.5)	0.81 (0.46–1.42)	0.79 (0.24–2.64)
Father’s education level				
High school above	54	38 (70.4)	1.00 (ref)	1.00 (ref)
High school	32	26 (81.3)	1.17 (0.71–1.92)	1.07 (0.54–2.10)
Junior school	37	29 (78.4)	1.28 (0.79–2.07)	1.07 (0.40–2.83)
Less than junior school	7	3 (42.9)	0.41 (0.13–1.33)	0. 19 (0.04–0.98)
Mother’s education level				
High school above	40	29 (72.5)	1.00 (ref)	1.00 (ref)
High school	30	23 (76.7)	1.02 (0.59–1.77)	0.90 (0.43–1.88)
Junior school	34	27 (79.4)	1.20 (0.71–2.04)	1.13 (0.50–2.56)
Less than junior school	26	17 (65.4)	0.91 (0.50–1.66)	1.34 (0.53–3.43)
Smoking				
No	123	91 (74.0)	1.00 (ref)	1.00 (ref)
Yes	7	5 (71.4)	0.98 (0.40–2.40)	1.44 (0.47–4.42)
Drinking				
No	90	67 (74.4)	1.00 (ref)	1.00 (ref)
Yes	40	29 (72.5)	1.03 (0.66–1.59)	0.76 (0.42–1.37)
Regular exercise				
No	51	36 (70.6)	1.00 (ref)	1.00 (ref)
Yes	79	60 (76.0)	1.29 (0.86–1.96)	1.34 (0.81–2.22)
Poor sleep quality				
No	115	87 (75.7)	1.00 (ref)	1.00 (ref)
Yes	15	9 (60.0)	0.61 (0.31–1.21)	0.41 (0.18–0.92)
High psychological stress				
No	105	78 (74.3)	1.00 (ref)	1.00 (ref)
Yes	25	18 (72.0)	0.97 (0.58–1.62)	1.16 (0.63–2.15)
Type of warts				
Common wart only	63	41 (65.1)	1.00 (ref)	1.00 (ref)
Plantar wart only	58	48 (82.8)	1.49 (0.98–2.27)	1.35 (0.83–2.20)
Other^b^	9	7 (77.8)	1.44 (0.65–3.22)	1.28 (0.51–3.27)
Number of warts				
Single	74	51 (68.9)	1.00 (ref)	1.00 (ref)
Multiple	56	45 (80.4)	1.45 (0.97–2.16)	1.28 (0.81–2.03)
Treatment				
No	78	54 (69.2)	1.00 (ref)	1 .00 (ref)
Yes	52	42 (80.8)	1.38 (0.92–2.07)	1.35 (0.83–2.18)

^a^Mutually adjusted for all other variables in the table. ^b^Other types include common as well as plantar wart, common as well as plane wart, and plane wart only. HR = hazard ratio.

## Discussion

In this study, we found that 1.4% of college students were affected with warts on their hands and/or feet. The majority of patients had only one wart on the hand or foot and the majority of warts were <1 cm in diameter. Slightly over half of patients experienced spontaneous resolution of warts within two years after the initial survey. Patients’ age, sleep quality, and paternal education level were shown to be independently associated with the resolution of warts.

In comparison to the studies that had involved young adults from the UK and the USA^14,15^, the overall prevalence rate of cutaneous warts in our study was lower than that in the UK study, while being similar to the US study^15^. In our survey, the prevalence of cutaneous warts varied slightly across different age groups: 1.4%, 1.3%, and 1.6% in those aged 14 to 20, 21 to 25, and 26 to 35 years, respectively. By contrast, the prevalence of warts in children and adolescents varied considerably from 0.3% to 8.6% in individuals aged 1 to 17 years, with peak prevalence occurring at 9 to 10 years^13^. The reasons for the different magnitudes of variation in cutaneous wart prevalence between children and college students remain largely unknown, which may possibly be attributed to age-related differences in physical activity and sports participation, the resistance of the stratum corneum, and host immunity.

The UK study documented no significant gender differences in prevalence of warts in adults^14^, but the US study showed that the warts were more frequent in male than female adults^15^. Consistent with the US study, the prevalence of warts was significantly higher in males in our study. After adjustments for various demographical and clinical factors, gender-specific differences remained statistically significant, suggesting that male gender might be an independent risk factor of cutaneous warts in our population. This may be attributed to males being physically more active compared to females^17^ and therefore at a higher risk of damage to the stratum corneum that serves as an entry point of HPV^18^. In this study, we also found that the prevalence of warts was negatively associated with fathers’ education. This may be partially due to that students whose fathers have a lower education level were more likely to come from families with relatively lower socioeconomic status, which has been reported to be a risk factor for the warts^9,14^. Notably, we could not exclude the possibility that the association of male gender and father’s education with the warts was an incidental finding, because we have made multiple sets of statistical comparisons regarding the differences of prevalence of warts among subgroups of students.

To the best of our knowledge, this is the first study to document the clinical characteristics of cutaneous warts in college students. We found that the percentage of patients with multiple warts (36%) or with warts ≥1 cm in diameter (4%) was substantially lower than that in children (43% and 37%, respectively)^16^. We also found that pain was the most commonly reported inconvenience (12%) in our patients, while in pediatric patients the percentage of pain was much lower (8%)^16^. Unsightly appearance, as the most commonly reported inconvenience (14%) in pediatric patients^16^, was only reported by 2% of our patients. Despite the aforementioned differences in clinical characteristics, the percentage of our patients who had sought treatment prior to the survey was comparable to that of pediatric patients (23% *vs*. 24%^10^).

The spontaneous resolution rate of warts within 1 and 2 years was reported to be 50% and 67% in children, respectively^16,19^. By contrast, less than one-third and slightly over half of our patients recovered spontaneously within 1 and 2 years after the initial survey, respectively, suggesting a lower likelihood of spontaneous resolution of warts in young adults compared to children. It has been reported that age and ethnicity may play a role in the resolution of warts in children^16^. The current study shows that age, sleep quality, and father’s education level may influence the resolution of wart in young adults; speculatively, this may be attributed to individual factors (such as immunity) and family socioeconomic status. In addition, we found that the type and number of warts did not seem to influence the outcome in young adults, which is in agreement with the findings in children^16^.

The present study has several strengths. First, to our knowledge, this is the first study focusing on epidemiological and clinical characteristics of cutaneous warts in college students. Second, the participation rate was high given that the survey was conducted along with the college-entry health examinations. Third, all participants were examined one by one for the presence of warts by trained dermatologists according to uniform diagnostic criteria. Fourth, we identified that several factors including patients’ age, sleep quality and father’s education level were independently associated with the resolution of warts, which provides new insights into the consultation and treatment concerning cutaneous warts. This study also has limitations. First, we only examined the hands and feet. However, this was less likely to result in significant underestimation of the overall prevalence of warts, as cutaneous warts on other parts of the body accounted for <4% of all warts^20^. Second, the patients were only followed up once, 2–3 years after the initial survey, and some participants could only provide an estimated time of resolution. Third, we did not collect information about hygienic practice and grooming of skin, which may influence the occurrence of the warts. Finally, our survey was only conducted in 3 colleges in Beijing, which may therefore limit the generalization of the findings.

In summary, this study provides an updated estimate of the prevalence of cutaneous warts as well as new insights into the clinical and prognostic profiles of cutaneous warts in college students, likely benefiting to the medical consultation and treatment concerning cutaneous warts in young adults.

## Methods

### Study Design and Participants

This study was a cross-sectional survey among the first-year undergraduate and postgraduate students (September 2012, March 2013, and September 2013) of three colleges in Beijing, and those diagnosed with warts were followed up between December 2015 and March 2016. This study protocol was approved by the Institutional Review Board of 306 Hospital of PLA, and all methods were performed in accordance with relevant guidelines/regulations; oral informed consent was obtained from all the participants before the survey.

### Procedure and Wart Examination

Along with the college-entry health examinations, the students were invited to complete a questionnaire containing questions about demographic information including age, sex, ethnicity, residential region, parents’ occupation, and education level. Then, the hands and feet of the students were examined one by one, following uniform diagnostic criteria, by dermatologists who were trained specifically prior to the start of the study. If a student was diagnosed with wart(s), the dermatologists collected detailed information via a face-to-face interview about the clinical characteristics of warts and potential influencing factors. The clinical characteristics included type, location, number, size, and duration of warts, duration from wart onset to seeing a doctor, suspected predisposing factors of warts, discomfort caused by warts, symptoms. The potential influencing factors of warts included smoking (yes or no), drinking (yes or no), regular exercise (yes or no), poor sleep quality (yes or no), and high psychological stress (yes or no). Smoking was defined as at least one cigarette per day. Dinking was defined as intake of at least 50 ml of Chinese liquor, 250 ml of wine, or 500 ml of beer per week. Regular exercise, sleep quality and psychological stress were self-reported by students based on their own perception. Two-three years later, a trained dermatologist revisited the patients on site to conduct a follow-up interview. In case the patients could not be reached on site, a telephone interview was conducted. During the follow-up interview, information on resolution of warts and types of topical treatment received between the initial survey and the follow-up visit was collected. Complete resolution was defined as no warts being visible and not palpated by hand. With regards to the types of treatments, the dermatologists responsible for the follow-up interview chose among the following: cryotherapy, CO_2_ laser, photodynamic therapy, pulsed dye laser, plasters containing 78% salicylic acid and 4% phenol, traditional Chinese medicine, interferon-α, retinoids, antitumor drugs and other treatments.

### Statistical Analyses

The chi-squared test was used to examine the statistical differences in the prevalence of warts between subgroups defined by demographic characteristics. Multivariate logistic regression analyses were performed to determine which factors might be independently associated with warts, through which the adjusted odds ratio (aOR) with 95% CI was calculated. Univariate and multivariate Cox proportional hazards models were used to estimate the hazard ratios for the resolution of warts. We used a complete case strategy in the multivariate analyses given that missing data on covariates was much lower (<5%). Statistical analyses were performed with IBM SPSS version 20.0. All statistical tests were two-sided using a significance level of alpha = 0.05.

## Data Availability

The datasets generated and/or analysed during the current study are available from the corresponding author on reasonable request.
